# Comparison of High Ligation Versus Low Ligation of the Inferior Mesenteric Artery (IMA) on Short-Term and Long-Term Outcomes in Sigmoid Colon and Rectal Cancer Surgery: A Meta-analysis

**DOI:** 10.7759/cureus.39406

**Published:** 2023-05-23

**Authors:** Ibrahim Reyaz, Nafisa Reyaz, Qais M Salah, Talwinder K Nagi, Abdur Rehman Mian, Ali Hanif Bhatti, Kinan Obeidat, Shamsha Hirani

**Affiliations:** 1 Internal Medicine, Christian Medical College and Hospital, Ludhiana, IND; 2 Medicine, Jawaharlal Nehru Medical College and Hospital, Aligarh, IND; 3 Internal Medicine, Faculty of Medicine, Al Quds University, Jerusalem, PSE; 4 Internal Medicine, Florida Atlantic University Charles E. Schmidt College of Medicine, Boca Raton, USA; 5 Internal Medicine, Caribbean Medical University, Willemstad, CUW; 6 Internal Medicine, Akhtar Saeed Medical and Dental College, Lahore, PAK; 7 Internal Medicine, University of Texas Medical Branch at Galveston, Galveston, USA; 8 Cardiology, Baqai Hospital, Karachi, PAK

**Keywords:** rectal cancer, sigmoid colon cancer, inferior mesentric artery, high ligation, low ligation

## Abstract

This study was done to compare the perioperative outcomes and long-term outcomes between low ligation and high ligation of the inferior mesentric artery (IMA) in sigmoid colon and rectal cancer surgery. This study was conducted following the recommendations of the Preferred Reporting Items for Systematic Review and Meta-Analyses (PRISMA). A literature search was performed in electronic databases including PubMed, CINAHIL, EMBASE, and Web of Science to identify studies published between January 1, 2015, and April 30, 2023. The outcomes assessed in this meta-analysis included postoperative complications (anastomotic leakage, surgical site infection, and postoperative ileus), intraoperative outcomes (duration of surgery in minutes, total intraoperative blood loss in milliliters, total lymph nodes harvested, and total number of metastatic lymph nodes), recovery outcomes (time to first flatus and length of hospital stay), and long-term outcomes (five-year mortality rate and disease-free survival rate). A total of 17 studies were included in this meta-analysis. Of these, six were randomized control trials (RCTs) and 11 were retrospective cohort studies. This meta-analysis suggests that lower ligation may be associated with a lower risk of anastomotic leakage compared to higher ligation in patients undergoing colon cancer surgery. However, there was no significant difference between the two techniques in terms of surgical site infection, postoperative ileus, total lymph nodes harvested, number of metastatic lymph nodes, duration of surgery, intraoperative blood loss, and length of hospital stay. Time to first flatus was significantly shorter in patients who underwent lower ligation. Additionally, there were no significant differences in the five-year mortality rate and disease-free survival rate between the two techniques. The results of this study indicate that both techniques are comparable in most aspects and suggest that the choice of technique should be based on individual patient factors and surgeon preference.

## Introduction and background

Colorectal cancer is a major cause of death globally, with rectal cancer accounting for over 30% of all cases [1]. It is among the most prevalent cancers worldwide and is responsible for the second-highest number of cancer-related deaths [2]. The two most common types of colorectal cancer are rectal cancer and sigmoid colon cancer [3]. The mainstay treatment for stages I-III of colorectal cancer is surgery, with curative surgery being the preferred approach. Two techniques used during surgery include high ligation and low ligation. High ligation involves tying off the inferior mesenteric artery (IMA) at its root, while low ligation is performed distal to the bifurcation of the left colic artery and is still a matter of debate for rectosigmoid cancer [4-5].

Performing high ligation of the IMA is a more straightforward procedure than low ligation. It also allows for maximum mobility of the left colon segment, which helps to prevent anastomotic tension. However, high ligation has two drawbacks [6]. Firstly, the remaining colon segment only receives blood from the superior mesenteric artery, which leads to reduced blood supply in the marginal artery. This lack of blood supply can be problematic, especially in patients with arteriosclerosis, who may be at an increased risk of anastomotic leakage [7]. The efficacy of high ligation in reducing cancer recurrence and improving survival rates has been called into question because the incidence of lymph node metastasis at the IMA root is relatively low [8]. Secondly, performing high ligation carries the risk of damaging the autonomic nerve plexus, which could delay recovery of bowel function and potentially impair genitourinary function [9].

Due to the ongoing debate surrounding the optimal technique for IMA ligation, previous studies have investigated the impact of each approach on patient outcomes. According to a review conducted by Hajibandeh et al. [10], there was no discernible difference between high ligation and low ligation with respect to the anastomotic leakage rate, the total number of harvested lymph nodes, or overall survival rates. Since the last meta-analysis conducted by Yin et al. [9], comparing high ligation and low ligation, certain new studies have been conducted. Therefore, we have performed an updated meta-analysis to compare the perioperative outcomes and long-term outcomes between low ligation and high ligation of the IMA in sigmoid colon and rectal cancer surgery.

## Review

Methodology

This study was conducted following the recommendations of the Preferred Reporting Items for Systematic Review and Meta-Analyses (PRISMA). A literature search was performed in electronic databases including PubMed, CINAHIL, EMBASE, and Web of Science to identify studies published between January 1, 2015, and April 30, 2023. The search terms used were "high ligation," "low ligation," "rectal cancer," "sigmoid colon cancer," and "inferior mesenteric artery." Additionally, the reference lists of included articles were manually searched to identify any overlooked articles during the initial search. The search was restricted to studies published in the English language.

Eligibility Criteria

The inclusion criteria for this meta-analysis were studies comparing low and high ligation of the IMA during curative resection of sigmoid colon or rectal cancer, regardless of the surgical approach (robotic, laparoscopic, or open surgery). The studies must have reported at least one of the outcomes assessed in this meta-analysis. Review articles, case reports, and editorials were excluded. Studies without a control group were also not included in this meta-analysis. Two authors independently assessed articles obtained through the online database search for eligibility criteria, and any disagreement was resolved through discussion and consensus.

Data Extraction and Quality Assessment

Data from the included studies were extracted using a pre-designed form developed in Microsoft Excel. Data extracted from the included studies included author name, year of publication, study design, sample size, study population, and outcomes. One author extracted the data, and the second author cross-checked and entered it into RevMan for analysis. Quality assessment was performed independently by two authors using the Newcastle-Ottawa Scale for observational studies (NOS) and the Cochrane risk of bias assessment tool for randomized controlled trials (RCTs). The NOS evaluated studies based on the selection of participants, group comparability, exposure ascertainment, and patient outcomes. The Cochrane risk of bias assessment tool assessed seven domains, and each domain was classified as "high," "low," or "unclear" risk of bias.

Outcomes

The outcomes assessed in this meta-analysis included postoperative complications (anastomotic leakage, surgical site infection, and postoperative ileus), intraoperative outcomes (duration of surgery in minutes, total intraoperative blood loss in milliliters, total lymph nodes harvested, and total number of metastatic lymph nodes), recovery outcomes (time to first flatus and length of hospital stay), and long-term outcomes (five-year mortality rate and disease-free survival rate).

Statistical Analysis

Data analysis was performed using Review Manager (RevMan) Version 5.4.1 (The Cochrane Collaboration, The Nordic Cochrane Centre, Copenhagen, 2020). For categorical outcomes, the risk ratio (RR) was calculated with a 95% confidence interval (CI), and for continuous outcomes, the mean difference and 95% CI were calculated. A p-value less than 0.05 was considered significant. Heterogeneity was assessed using I-square statistics. In case of an I-square value less than 50%, a fixed-effect model was used; otherwise, a random-effect model was used. Subgroup analysis was performed based on the study design.

Results

**Figure 1:**
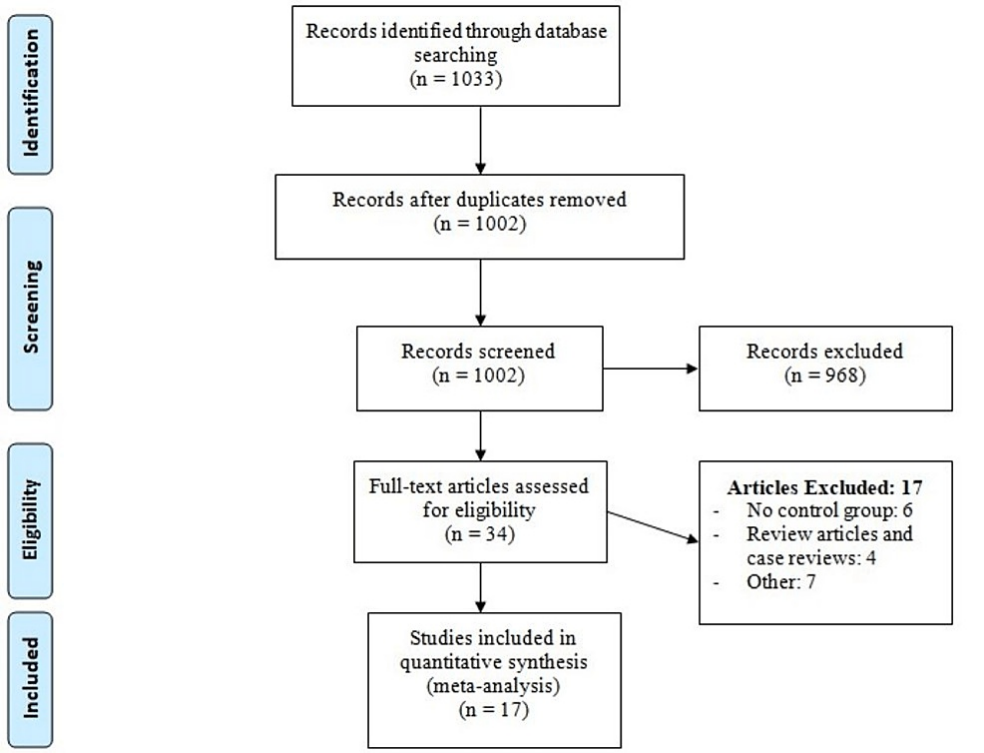
Process of study selection.

**Figure 2:**
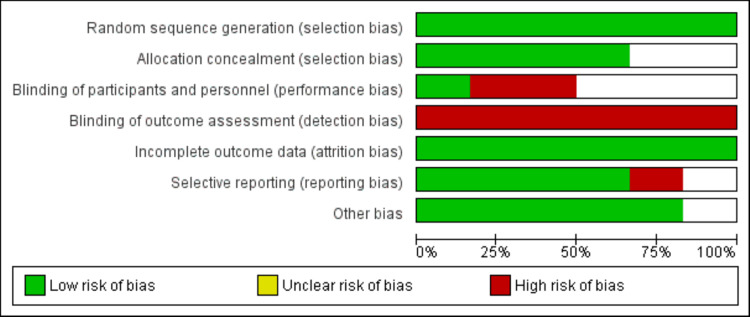
Risk of bias (quality assessment) graph of RCTs. RCTs, randomized control trials

Figure 1 shows the PRISMA flowchart of the process of study selection. Initial database searching yielded 1033 studies published between January 2015 and April 2023. After removing duplicates, 1002 studies were initially screened using titles and abstracts. Full-text of 34 studies was obtained and detailed evaluation of inclusion and exclusion criteria was done. A total of 17 studies were included in this meta-analysis. Of these, six were RCTs and 11 were retrospective cohort studies. The total number of patients was 4520, which included 2049 patients who received low ligation and 2471 who received high ligation. Table 1 shows characteristics of the included studies. Table 2 shows quality assessment of retrospective cohort studies. Figure 2 shows quality assessment of RCTs.

**Table 1:** Characteristics of included studies. RCT, randomized control trial; NR, not reported

Author	Year	Design	Tumor location	Surgery type	Groups	Sample size	Age (years)	Males (%)
Chen et al. [11]	2020	Retrospective cohort	Rectum	Laproscopy	High ligation	218	57.9 vs 58.6	54 vs 51.5
					Low ligation	216		
Crocetti et al. [12]	2019	Retrospective cohort	Sigmoid colon and rectum	Laproscopy	High ligation	65	63.4 vs 62.1	44.6 vs 49.1
					Low ligation	55		
Draginov et al. [13]	2020	Retrospective cohort	Sigmoid colon and rectum	Laproscopy	High ligation	158	67 vs 63	54.4 vs 59.3
					Low ligation	123		
Feng et al. [14]	2021	RCT	Rectum	Laproscopy	High ligation	47	60.5 vs 59.8	55.3 vs 50
					Low ligation	48		
Fujji et al. [15]	2018	RCT	Rectum	Laproscopy and open surgery	High ligation	164	65.9 vs 65.6	62.8 vs 60.1
					Low ligation	160		
Gomceli et al. [16]	2021	Retrospective cohort	Rectum	Robotic	High ligation	39	61.6 vs 62.5	64.1 vs 55.3
					Low ligation	38		
Guo et al. [17]	2017	RCT	Rectum	Laproscopy	High ligation	29	NR	NR
					Low ligation	28		
Hsu et al. [18]	2023	Retrospective cohort	Sigmoid colon and rectum	Laproscopy	High ligation	245	64.8 vs 64.1	57.6 vs 58.8
					Low ligation	245		
Kruszewski et al. [19]	2021	RCT	Sigmoid colon and rectum	Open surgery	High ligation	65	64 vs 65	57 vs 52
					Low ligation	65		
Lee et al. [20]	2018	Retrospective cohort	Sigmoid colon	Laproscopy	High ligation	51	66.1 vs 66.6	66.7 vs 71.1
					Low ligation	83		
Mari et al. [21]	2020	RCT	Rectum	Laproscopy	High ligation	101	NR	NR
					Low ligation	95		
Matsuda et al. [22]	2015	RCT	Rectum	Laproscopy	High ligation	51	69 vs 67	64.7 vs 69.1
					Low ligation	49		
Park et al. [23]	2020	Retrospective cohort	Sigmoid colon and rectum	Laproscopy	High ligation	613	62 vs 62	66.4 vs 65
					Low ligation	163		
Wang et al. [24]	2022	Retrospective cohort	Sigmoid colon and rectum	Laproscopy	High ligation	283	57.5 vs 58.5	64 vs 57
					Low ligation	307		
Yasuda et al. [4]	2016	Retrospective cohort	Sigmoid colon and rectum	NR	High ligation	42	64.5 vs 68	62 vs 63
					Low ligation	147		
You et al. [25]	2020	Retrospective cohort	Rectum	Laproscopy	High ligation	174	57.2 vs 58.1	67.2 vs 66.2
					Low ligation	148		
Zhang et al. [26]	2021	Retrospective cohort	Rectum	Laproscopy	High ligation	126	60.3 vs 61.3	50.8 vs 60.8
					Low ligation	79		

**Table 2:** Quality assessment of retrospective cohort studies.

Study Id	Selection	Comparison	Outcome	Overall
Chen et al. [11]	4	1	3	Good
Crocetti et al. [12]	4	1	3	Good
Draginov et al. [13]	4	1	2	Good
Gomceli et al. [16]	4	2	2	Good
Hsu et al. [18]	4	2	2	Good
Lee et al. [20]	2	2	3	Good
Park et al. [23]	4	1	2	Good
Wang et al. [24]	4	2	2	Good
Yasuda et al. [4]	4	1	3	Good
You et al. [25]	4	2	3	Good
Zhang et al. [26]	4	1	3	Good

Post-operative outcome

Anastomotic Leakage

**Figure 3:**
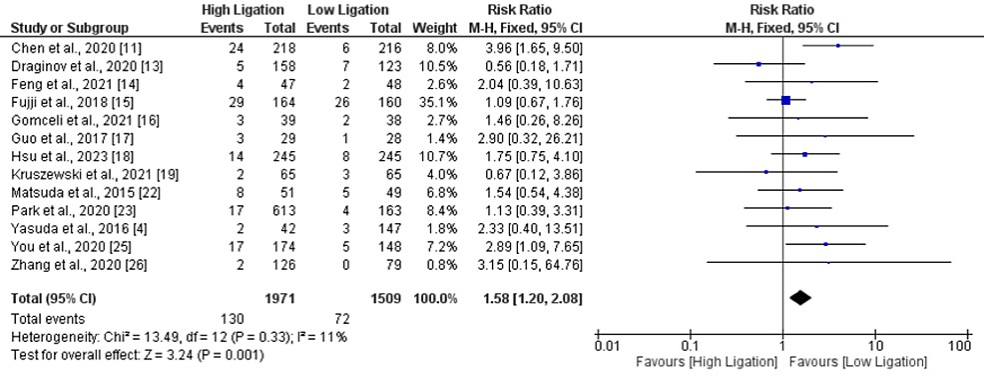
Anastomotic leakage. Sources: References [4, 11, 13-19, 22-23, 25-26]

A total of 13 studies assessed anastomotic leakage. The analysis revealed that the incidence of anastomotic leakage was significantly lower with lower ligation compared to patients with higher ligation (RR: 1.58, 95% CI: 1.20, 2.08) as shown in Figure 3. No significant heterogeneity was reported among the study results (I-square: 11%).

Surgical Site Infection

**Figure 4:**
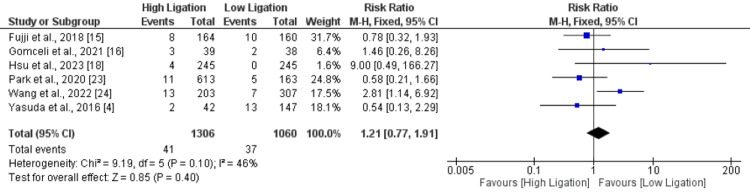
Surgical site infection. Sources: References [4, 15-16, 18, 23-24]

A total of six studies compared risk of surgical site infection between two groups. No significant difference was found between lower ligation group and higher ligation group in terms of surgical site infection (RR: 1.21, 95% CI: 0.77, 1.91) as shown in Figure 4. No significant heterogeneity was reported among the study results (I-square: 46%).

Postoperative Ileus

**Figure 5:**
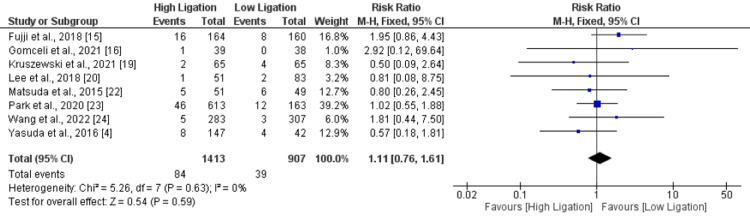
Postoperative ileus. Sources: References [4, 15-16, 19-20, 22-24]

A total of eight studies compared risk of postoperative ileus between patients in lower ligation groups and higher ligation groups. No significant difference was found between two groups in terms of risk of postoperative ileus (RR: 1.11, 95% CI: 0.76, 1.61) as shown in Figure 5. No significant heterogeneity was reported among the study results (I-square: 0%).

Surgical quality outcomes

Total Lymph Nodes Harvested

**Figure 6:**
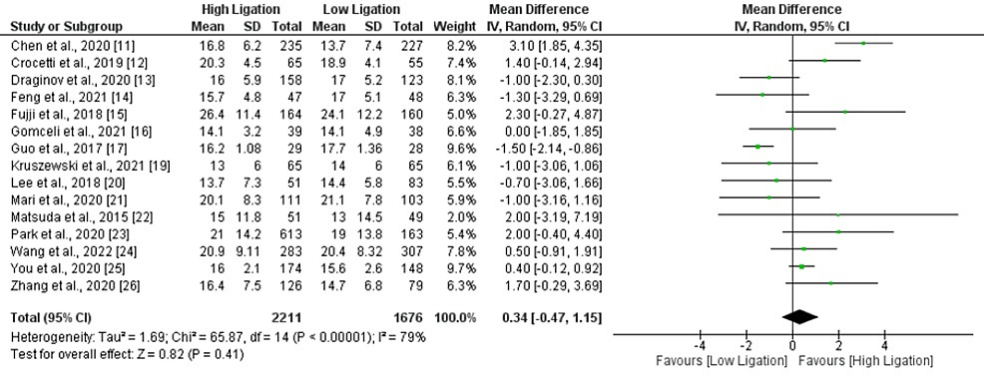
Total lymph nodes harvested. Sources: References [11-17, 19-26]

A total of 15 studies were included. No significant difference was found between two groups in mean number of total lymph nodes harvested (MD: 0.34, 95% CI: -0.47, 1.15) as shown in Figure 6. Significant heterogeneity was reported among the study results (I-square: 79%).

Number of Metastatic Lymph Nodes

**Figure 7:**
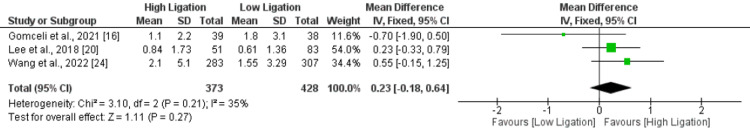
Metastatic lymph nodes. Sources: References [16, 20, 24]

In total three studies were included. No significant difference was found between two groups in mean number of metastatic lymph nodes (MD: 0.23, 95% CI: -0.18, 0.64) as shown in Figure 7. No significant heterogeneity was reported among the study results (I-square: 35%).

Duration of Surgery in Minutes

**Figure 8:**
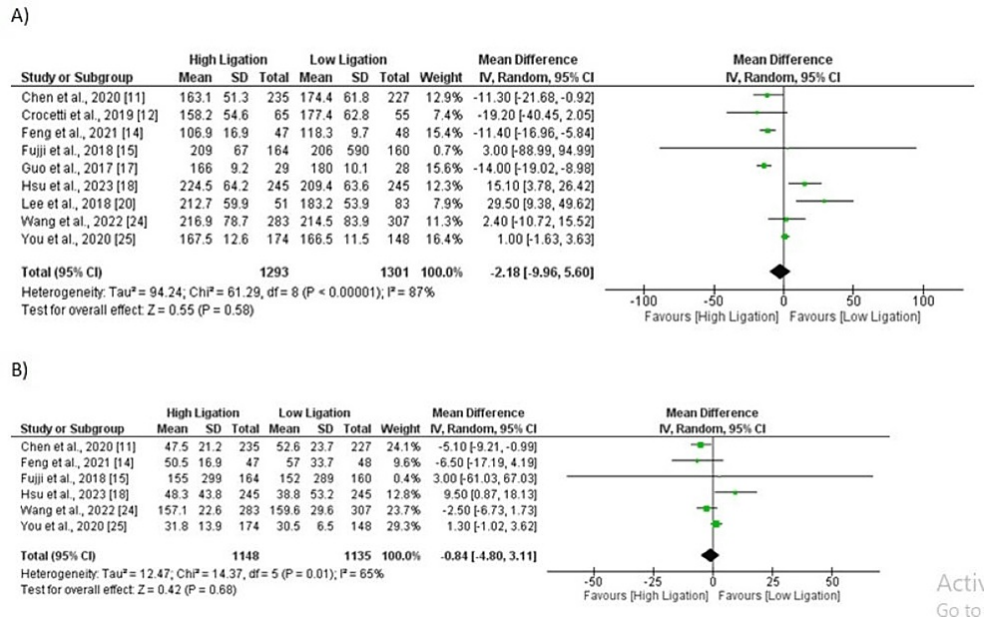
A) Duration of surgery; B) Intraoperative blood loss. Sources: References [11-12, 14-15, 17-18, 20, 24-25]

The results of the meta-analysis showed that there was no significant difference in the duration of surgery between the low ligation and high ligation groups. The pooled mean difference was -2.81 (95% CI: -9.96, 5.60), which was not statistically significant as shown in Figure 8A. The analysis was based on a total of nine studies, and the findings suggest that both low ligation and high ligation techniques are comparable in terms of the duration of surgery. Significant heterogeneity was reported among the study results (I-square: 87%).

Intraoperative Blood Loss

Based on the meta-analysis of six studies comparing the low ligation and high ligation techniques, there was no significant difference in the intraoperative blood loss between the two groups. The pooled mean difference was -0.84 (95% CI: -4.80, 3.11), indicating that the two techniques are comparable in terms of blood loss. as shown in Figure 8B. Significant heterogeneity was reported among the study results (I-square: 65%).

Post-operative recovery outcomes

Length of Hospital Stay (Days)

**Figure 9:**
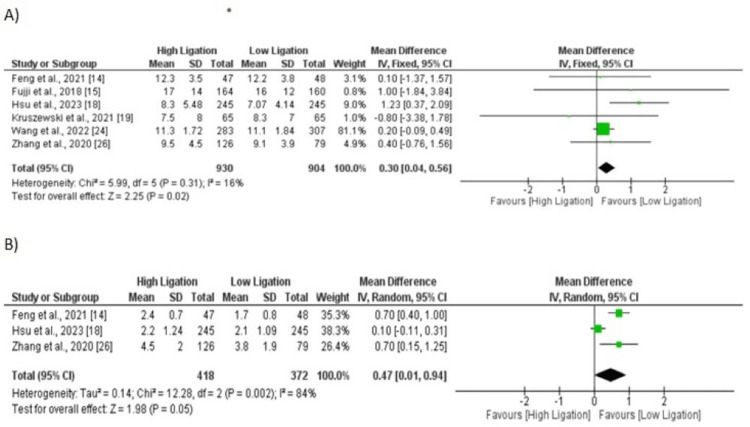
A) Length of hospital stay; B) Time to first flatus. Sources: References [14-15, 18-19, 24, 26]

In total six studies were included in the analysis of length of hospital stay. Mean length of hospital stay was significantly low in patients in lower ligation group compared to patients in higher ligation group (MD: 0.30, 95% CI: 0.04, 0.56) as shown in Figure 9A. No significant heterogeneity was reported among the study results (I-square: 16%).

Time to First Flatus (Days)

Three studies included in pooled analysis of comparing time to first flatus between two groups. Pooled analysis showed that time to first flatus was significantly higher in patients in high ligation group compared to its counterpart (MD: 0.47, 95% CI: 0.01, 0.94) as shown in Figure 9B. Significant heterogeneity was reported among the study results (I-square: 84%).

Long-term outcomes

Five-Year Mortality

**Figure 10:**
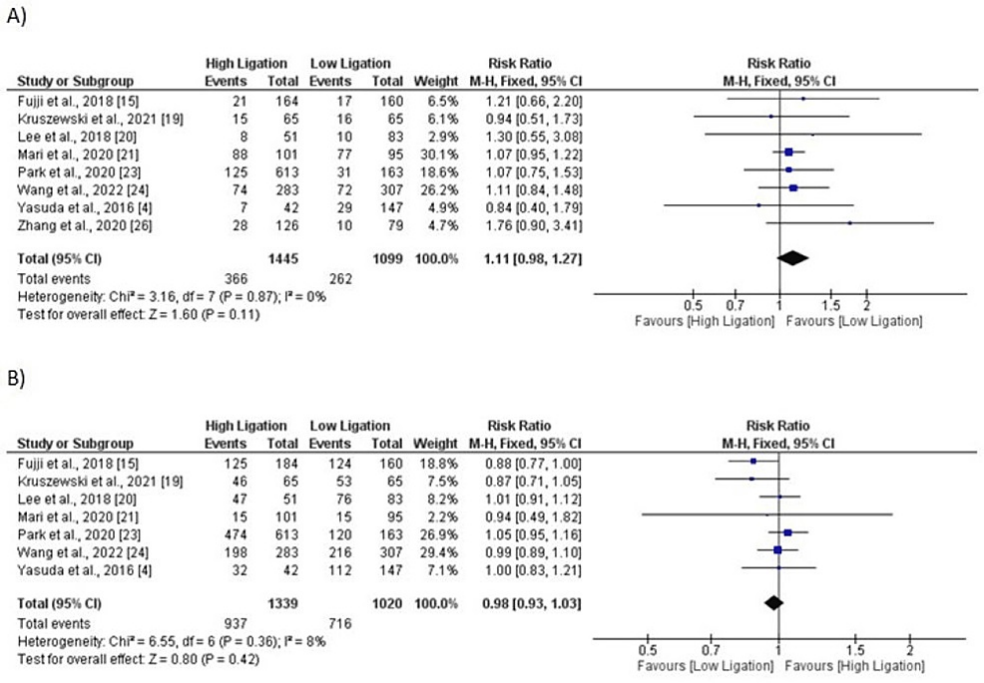
A) Five-year mortality; B) Five-year disease-free survival. Sources: References [4, 15, 19-21, 23-24, 26]

Eight studies compared mortality risk in five years in patients between two groups. No significant difference is reported in the pooled analysis in risk of five-year mortality between patients in low ligation group and patients in high ligation group (RR: 1.11, 95% CI: 0.97, 1.27) as shown in Figure 10A. No significant heterogeneity was reported among the study results (I-square: 0%).

Disease-Free Survival

Eight studies compared the rate of disease-free survival between patients in low ligation group and patients in high ligation group. The analysis revealed no significant difference in the disease-free survival rate between two groups (RR: 0.98, 95% CI: 0.93, 1.03) as shown in Figure 10B. No significant heterogeneity was reported among the study results (I-square: 8%).

Subgroup Analysis

Table 3 shows the results of the subgroup analysis. Regarding anastomotic leakage, the results were different in RCTs and retrospective cohort studies. The pooled analysis of RCTs did not report a significant difference between low ligation and high ligation in the rate of anastomotic leakage. The pooled analysis of RCTs reported different results compared to the pooled analysis of retrospective cohort studies in terms of five-year disease-free survival rate. Concerning the number of lymph nodes harvested, five-year mortality, and post-operative ileus, the results were consistent across both subgroups. However, considering the number of RCTs that assessed anastomotic leakage and the five-year disease-free survival rate, the findings need to be interpreted with caution.

**Table 3:** Results of subgroup analysis. ^Presented as mean difference (95% CI) *Significant at p-value < 0.05 RCT, randomized control trial; RR, risk ratio; CI, confidence interval

Outcome	Subgroups	Number of studies	RR (95% CI)	I-square (%)
Anastomotic leakage	RCT	5	1.22 (0.81, 1.82)	0
	Retrospective cohort study	8	2.02 (1.32, 3.10)*	25
Postoperative ileus	RCT	3	1.25 (0.69, 2.26)	31
	Retrospective cohort study	5	1.02 (0.63-1.66)	0
Number of lymph nodes harvested^	RCT	6	-0.70 (-1.79, 0.39)	47
	Retrospective cohort study	9	0.81 (-0.05, 1.68)	70
Five-year mortality	RCT	3	1.08 (0.92, 1.25)	0
	Observational	6	1.12 (0.92, 1.36)	0
Five-year disease-free survival	RCT	3	0.88 (0.79, 0.98)*	0
	Retrospective cohort study	4	1.02 (0.96, 1.08)	0

Discussion

The present meta-analysis was conducted to compare low ligation and high ligation of the IMA in terms of early and late outcomes for sigmoid colon and rectal cancers. The meta-analysis concluded that long-term outcomes, including five-year mortality and disease-free survival, were similar in both groups, along with intraoperative outcomes. However, the rate of anastomotic leakage was significantly higher in the high ligation group compared to the low ligation group. A meta-analysis conducted by Yin et al. also reported a higher incidence of anastomotic leakage in the low ligation group [9]. However, by conducting a subgroup analysis based on study design, we found a slightly increased rate of anastomotic leakage in the high ligation group compared to the low ligation group, but the difference was statistically insignificant. None of the five RCTs reported significant differences between the two groups in terms of the rate of anastomotic leakage. The inconsistency between the RCTs and the retrospective studies might because the case number of the RCTs was relatively smaller than retrospective studies, which made the statistical significance hard to reach.

Anastomotic leakage is an important index of the success of gastrointestinal surgery. According to past studies, the incidence of colorectal or coloanal anastomosis ranged widely; well-experienced surgeons considered an anastomotic leakage rate between 3% and 6% acceptable [27]. Factors that impact anastomotic leakage are intricate. Some are unchangeable and linked to the patients themselves (for instance, being male, having diabetes, renal insufficiency, obesity, and malnutrition) or their tumors (such as being located distally, being bulky, and being in an advanced stage). Other causes are associated with treatments before surgery, such as preoperative radiotherapy or being treated with anti-vascular endothelial growth factor monoclonal antibodies [28]. The level at which the blood vessels are tied has often been identified as a possible contributing factor to anastomotic leakage, as it can potentially disrupt blood flow to the area where the intestine is reconnected in rectal surgery. Even though the marginal artery usually connects the superior mesenteric artery and the IMA, which provides significant collateral circulation to the bowel, there is a chance that the continuity of the marginal artery may be interrupted at Griffith's point in 5%-7% of people [29].

In terms of oncological perspective, our meta-analysis did not report any significant difference between high ligation and low ligation in terms of the total number of lymph nodes harvested and the number of metastatic lymph nodes. Past studies also reported that the number of lymph nodes harvested was not significantly different between the two study groups [30-31]. Additionally, no significant difference was found between the two groups in terms of five-year mortality and five-year disease-free survival. These findings were consistent with the past studies [9-10].

Few studies in the past reported longer operation time in the improved low ligation group, which reflected the enhanced operational complexity. Performing a complete dissection of apical lymph nodes around the root of the IMA while preserving the left colic artery (LCA) during laparoscopic rectal cancer surgery is a challenging task, especially for obese patients. The process of improved low ligation is more complex and requires more operation steps and skilled cooperation compared to high ligation. Additionally, the surgery becomes more complex due to the variation in the origin of the LCA from the IMA [32-33]. As surgical techniques have advanced, the time required for this particular step has been significantly reduced, leading to no statistically significant difference in operation time between the two groups in the current meta-analysis. Furthermore, the amount of blood loss during surgery was not significantly different between the two groups in our meta-analysis, as most of the surgical procedures were identical, except for the retention of the left colic artery.

The present meta-analysis has certain limitations. Firstly, moderate to high heterogeneity was reported among study results on certain outcomes were insurmountable in the present meta-analysis. However, we performed subgroup analysis based on the study design to compare pooled analysis of all-results with results obtained through combined analysis of RCTs. Secondly; there is currently no standardized approach for surgical details, specifically regarding the extent of D3 lymphatic clearance and the technique for preserving the superior hypogastric plexus (SHP). Lastly, we were not able to perform subgroup analysis based on certain characteristics including neoadjuvant therapy, tumor size, and so on. Therefore, in the future more RCTs are required with larger sample size to evaluate the effect of lower ligation and high ligation on perioperative outcomes. 

## Conclusions

In conclusion, this meta-analysis suggests that lower ligation may be associated with a lower risk of anastomotic leakage compared to higher ligation in patients undergoing colorectal cancer surgery. However, there was no significant difference between the two techniques in terms of surgical site infection, postoperative ileus, total lymph nodes harvested, number of metastatic lymph nodes, duration of surgery, intraoperative blood loss, and length of hospital stay. Time to first flatus was significantly shorter in patients who underwent lower ligation. Additionally, there were no significant differences in the five-year mortality rate and disease-free survival rate between the two techniques. The results of this study indicate that both techniques are comparable in most aspects and suggest that the choice of technique should be based on individual patient factors and surgeon preference. It is important to note that more high-quality randomized controlled trials are needed to confirm these findings and to determine the long-term outcomes associated with each technique.
